# Peroxidase activity in scutella of maize in association with anatomical changes during germination and grain storage

**DOI:** 10.1186/2193-1801-3-399

**Published:** 2014-08-02

**Authors:** José Isaac Corona-Carrillo, Mitzi Flores-Ponce, Gerardo Chávez-Nájera, David Manuel Díaz-Pontones

**Affiliations:** Department of Health Sciences, Division of Biological and Health Sciences, Laboratory for Tissue Biochemistry, Universidad Autónoma Metropolitana-Iztapalapa, Mexico City, Mexico; Posgrado en Biología Experimental, Division of Biological and Health Sciences, Universidad Autónoma Metropolitana-Iztapalapa, Mexico City, Mexico

**Keywords:** Maize (*Zea mays* L.), Fibrous layer, Controlled deterioration, Epidermis, Scutellum, Peroxidase

## Abstract

The embryo of the maize grain (*Zea mays* L.) is separated from the starchy endosperm by a fibrous structure, which is called the fibrous layer (FL). Using histochemical staining, it was determined that the FL is composed of collapsed cellular layers that contain phenols, neutral lipids, and 1,3-β-glucan. Due to its composition, the FL prevents free diffusion and separates the embryo from the endosperm during germination. Twenty-four hours after imbibition, the scutellum epidermis initiated a series of asynchronous spatial modifications, including cell growth, the perforation of cell walls, increased peroxidase activity in the apoplastic space, and elevated levels of superoxide, phenols, and other components that interact with the fibrous layer, enabling its transformation in addition to the free flow between compartments. During storage at high relative humidity levels, which leads to fast or slow deterioration depending on the temperature, the activity of phenol peroxidase in the scutellum was associated with a loss of vigor and reduced germination capacity when compared with low temperature and low relative humidity conditions. Such deterioration is associated with alterations in autofluorescent emissions from endogenous compounds in the scutellum, indicating changes in the microenvironment or in the differential proportions of epidermal and FL components.

**Electronic supplementary material:**

The online version of this article (doi:10.1186/2193-1801-3-399) contains supplementary material, which is available to authorized users.

## Background

Maize is one of the most important crops in the world along with wheat and rice because it is used as human and cattle food and as raw material for industrial purposes (Consonni et al. 2005). Creole maize is a variety that has been generated from Prehispanic times in the central region of Mexico, which has not been fully characterized. It is a material that has been cultivated and has adapted to the conditions of Mexico, preserving diversity and genomic variability that gives to maize an ability to adapt to different environmental conditions and facilitated their dispersion. Therefore the study of these creoles maizes allows to know the common morphological-functional characteristics that after domestication have been preserved, so far some of them are unknown, and may be key factors in development, germination and seedling establishment. These features must be taken into account for future plans of phytoimprovement.

In cereals, the endosperm is a persistent tissue (Sabelli and Larkins 2009) that works as an embryonic appendix whose primary function is the storage of carbohydrates, proteins, lipids, and minerals that feed the embryo following germination (Berger 1999). The maize endosperm is composed of the basal endosperm transfer layer, the embryo surrounding region, and the central starchy endosperm (SE), which includes the aleurone (Scanlon and Takacs 2009). For barley grain, it has been reported that the SE adjacent to the embryo consists of a layer of multistratified cells. During grain ripening, these cells are compressed and tightly packed together, while the few starch granules that are contained in the cell layer are degraded. This layer of cells was described as the crushed cell layer by MacGregor and Dushnicky (1989). The presence of a cell layer that is similar to the crushed cell layer has been reported in maize. Enríquez-Arredondo et al. (2005) found a layer with a fibrous appearance that is located between the scutellum and the endosperm in embryos of the maize landrace Montecillos A6♀2. Additionally, a similar structure that is located in the fibrous layer (FL) region has been described pictorially (Figure twenty one in Kiesselbach and Walker 1952; Figure one A in Tnani et al. 2011). However, these authors made no direct reference to the mentioned structure, and no specific function was assigned to it.

The embryo is formed by two structures: the embryonic axis and scutellum. The scutellum acts as a reserve that secretes, absorbs, and transfers nutrients (Dolfini et al. 2007). It is composed of epidermal cells, parenchymal cells, and components of the vascular system (Enríquez-Arredondo et al. 2005). Following germination, the epidermal cells are elongated towards the surface of the structure, and spaces appear between them, turning them into finger-like extensions that grow towards the SE. These modifications are typical of an epithelium (Bewley and Black 1994; Szcziparev 2006; Tnani et al. 2011).

The maize scutellum shows phenol peroxidase activity (PODa) in the basal area of the surface of the scutellum, in the root vascular cylinder, and in the aleurone layer (García-Lara et al. 2007). Class III plant peroxidases (EC 1.11.1.7) are a group of apoplastic and plasma membrane-bound enzymes that are monomeric, N-glycosylated, and contain a heme group that catalyzes the reduction of H_2_O_2_. Peroxidases receive electrons from various reducing donors that accumulate in most seeds, including phenolic groups, such as lignin precursors and flavonoids (Hiraga et al. 2001; Passardi et al. 2005; Winkel-Shirley 2002). These compounds act as antioxidants and are involved in latency, germination, and viability (Pourcel et al. 2007; Winkel-Shirley 2002). A wide range of physiological functions have been assigned to peroxidases, such as the cross-linking of phenolic compounds to proteins and polysaccharides (Fry 2004; Hollmann and Arends 2012), the deposit of polyphenols (Lagrimini 1991), and suberization (Bernards and Lewis 1998). Peroxidases play a dual role, functioning either as antioxidants, in which they consume H_2_O_2_ using a reduced molecule, or as pro-oxidants, in which they produce reactive oxygen species (ROS), such as O_2_^.-^ or °OH, via oxidative and hydroxylic cycles, respectively (Liszkay et al. 2003).

ROS are molecules that are produced continuously in cells by normal metabolism during development, germination, and the storage of seeds. They play a dual role, acting either in signaling pathways or as toxic products that accumulate due to stress (Apel and Hirt 2004). Therefore, their production must be finely regulated. Due to their reactivities, ROS can oxidize all types of cellular components (Møller et al. 2007); however, when their concentrations are regulated within a specific window (Bailly et al. 2008), they participate in diverse favorable processes, such as cellular elongation, which is caused by the modification and wall-loosening as a result of the hydrolysis of polysaccharide bonds by °OH (Schopfer 2001). When ROS concentrations exceed the oxidative window, stress is generated. This may compromise seed viability, for example, during storage (Goel and Sheoran 2003). Excessive ROS concentrations are also associated with decreased PODa in various seeds, including sunflower, soybean, pear stock, wheat, cotton and radish (Balešević-Tubić et al. 2005, 2011; Bao et al. 2011; Chauhan et al. 2011 Goel and Sheoran 2003; Scialabba et al. 2002).

The loss or reduction of germination capacity is one of the primary problem that is faced by farmers. It has been proposed that seeds have the highest vigor during physiological ripeness, which decreases slowly during storage (Goel and Sheoran 2003). Various factors affect seed viability during events that precede sowing, such as the degree of maturity, state of seed development, and mechanical damage that is caused during harvesting (Eskandari 2012) or storage. The loss of seed viability is an inevitable consequence of storage and depends primarily on the temperature and relative humidity of the storage conditions, the moisture content of the seed, the duration of storage, and the type and initial quality of the seeds (Daniel 2007).

The FL is strategically positioned between the scutellum and endosperm. Accordingly, the objective of this work was to analyze the chemical composition of the FL and its ability to generate two separate compartments: the embryo from the endosperm. At the end of germination, which is characterized by the protrusion of the radicle, we assessed the transformations that occurred in the epidermal cells of the scutellum, the spatial correlation of peroxidase activity, and modifications in the FL that paralleled changes in permeability. Likewise, we evaluated the deterioration of the embryo and corresponding alterations that occurred during storage, the epidermal cells of the scutellum, the FL and peroxidase activity following germination.

## Results

### Chemical composition of fibrous layer

**Figure 1:**
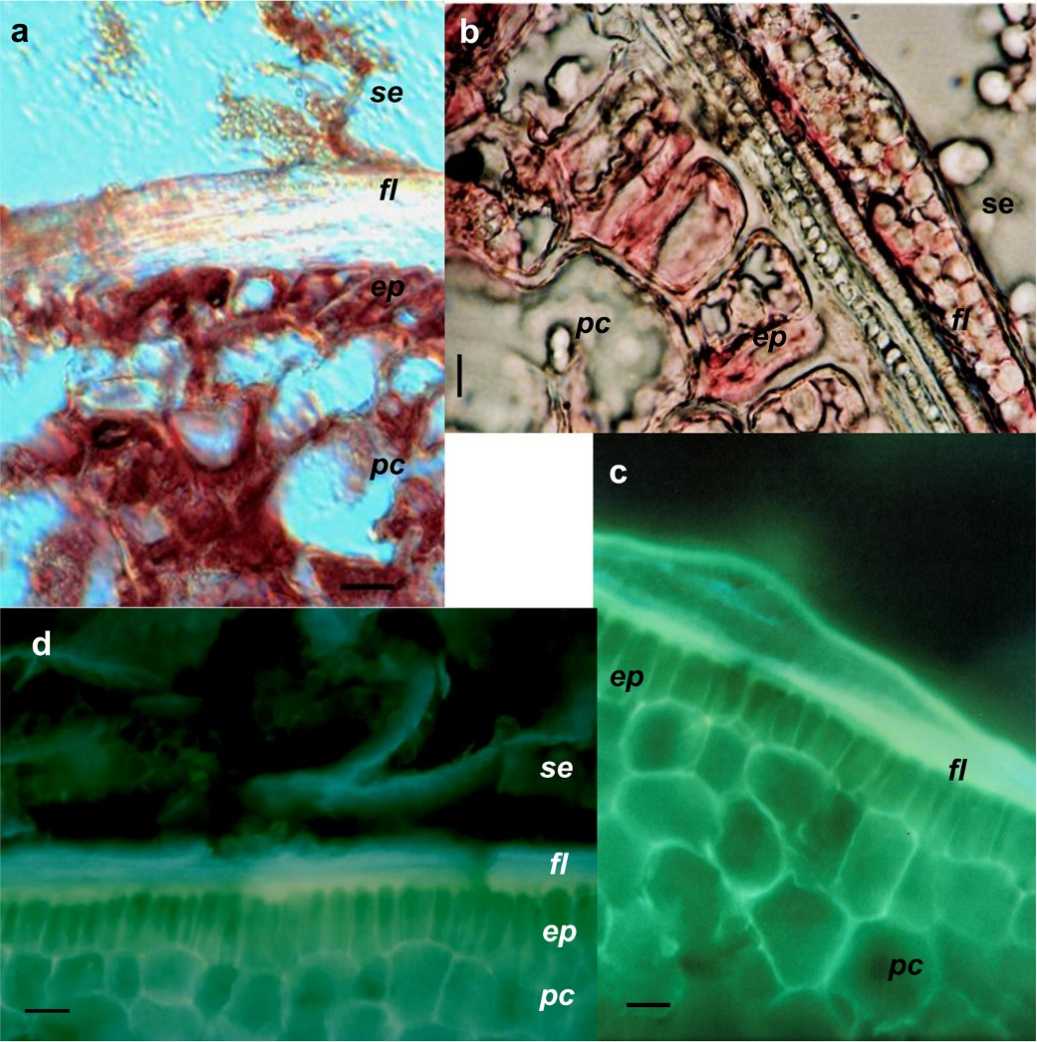
**Chemical composition of fibrous layer. a**, location of lipids detected by Oil Red O; **b**, location of lipids detected by Sudan IV; **c**, fluorescence of phenols enhanced with diphenylborinic acid; **d**, presence of β-glucan of the callose type identified by aniline blue. Abbreviations: *ep*, epidermal cells; *fl*, fibrous layer; *pc*, parenchyma cells; *se*, central starchy endosperm. Bar: a-d, 50 μm.

The epidermis of the scutellum in a mature embryo is constituted of a monostratified layer of cuboidal cells (Figure 1a-d). Enveloping the scutellum is the FL, which is in intimate contact with the epidermal cells of the scutellum and generates the border of the SE as previously reported by Enríquez-Arredondo et al. (2005). The epidermal cells, the parenchyma of the scutellum, and the SE remnants show intense red staining with Oil Red O (Figure 1a) and Sudan IV (Figure 1b), indicating the presence of lipids, as expected due to their storage functions (Szcziparev 2006; Tan and Morrison 1979). Lipids are also visible in the FL region, although they are less prominent. The greatest level of fluorescence was detected in the FL due to the presence of phenolic compounds (Figure 1c), whereas the phenols were widely distributed on the walls of the parenchyma and the epidermal cells of the scutellum. Aniline blue, which detects β-glucanes of the callose type, showed bright blue fluorescence in the FL in contrast with the white fluorescence that was observed of the cells from the epidermis and parenchyma (Figure 1d).

### Permeability of fibrous layer

**Figure 2:**
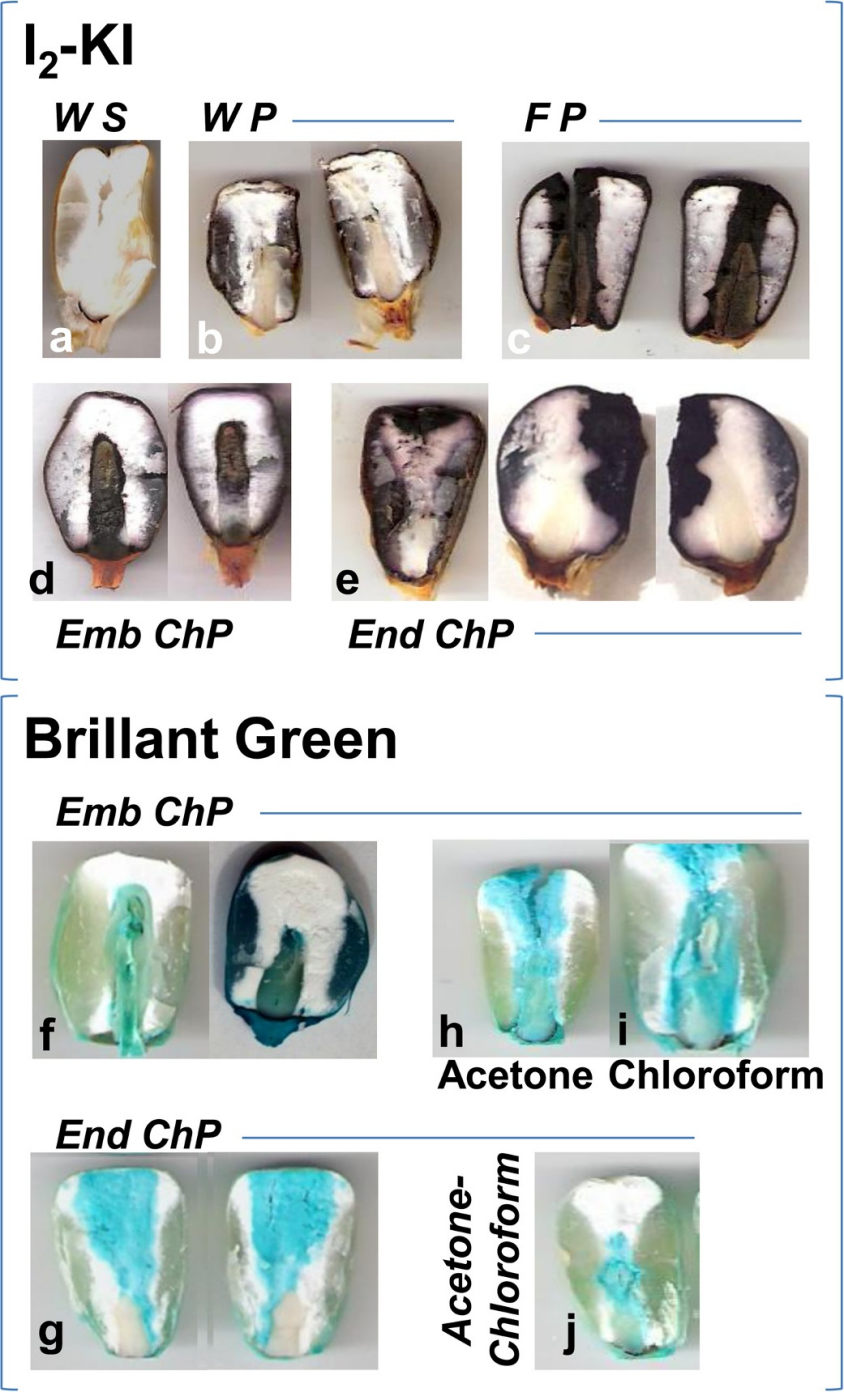
**Diffusion through fibrous layer.** Dry grains were perforated through the embryo or endosperm, and the diffusion of aqueous or ethanol dyes was assessed; **a**, grain without stain; **b**-**e**, diffusion of I_2_-KI; **f**-**j**, diffusion of Brilliant Green; **b**, grain without perforation; **c**, grain perforated through both compartments; **d** and **f**, perforation through embryo; **e** and **g**, perforation through endosperm; **h** and **i**, grains perforated through endosperm, and **h**, treated with acetone, or **i**, treated with chloroform; **j**, grains perforated through embryo and treated with acetone:chloroform. Abbreviations: *WS*, without stain; *WP*, without perforation; *FP*, full perforation; *Emb ChP*, embryo chamber perforation; *End ChP*, endosperm chamber perforation.

Under native conditions, a longitudinal cut of a dry grain of maize showed a yellow coloration in the pericarp, white in the endosperm, and pale yellow in the scutellum-embryonic axis (Figure 2a). This allowed for the visual contrast of regions during the assay and the examination of stain diffusion through the FL. In those grains where structural integrity was maintained, an external brown staining of the pericarp by I_2_-KI was evident without penetration of the dye, and the endosperm and embryo displayed their natural colors (Figure 2b). Perforation through the embryo or endosperm without causing damage to the FL enabled the diffusion of I_2_-KI or Brilliant Green, which stained either the embryo (Figure 2d, 2f) or the endosperm (Figure 2g) exclusively, depending on which of the two structures had been punctured. The diffusion speed of I_2_-KI in the endosperm was slower than that in the embryo, indicating a lower diffusion index. However, when the dye came into contact with the FL, it diffused preferentially, surrounding the FL through the endosperm without penetrating the embryo, indicating the existence of a barrier to diffusion between the two structures (Figure 2e). When the grains were perforated from the embryo through the FL, I_2_-KI diffused freely, staining the embryo and the endosperm (Figure 2c). Grains that were perforated from the endosperm and then treated with acetone (Figure 2h) or chloroform (Figure 2i) and those that were perforated from the embryonic chamber and treated with acetone:chloroform (Figure 2j) showed the free diffusion of Brilliant Green between the two organs, independent of the perforation site.

These results imply the following with respect to dry grains: 1) the integrity of the FL prevents the free diffusion of aqueous or ethanol-based dyes beyond the point of administration; 2) ethanol, which was the solvent that was used for the Brilliant Green dye, does not alter the structure of the FL within the time frame of the present study; and 3) treatments with acetone, chloroform, or an acetone:chloroform mixture allows for the removal of non-polar components that are present in the FL, thus favoring the diffusion of the ethanol-based stain between the two compartments.

### Effect of imbibition on morphology of epidermal cells of scutellum

**Figure 3:**
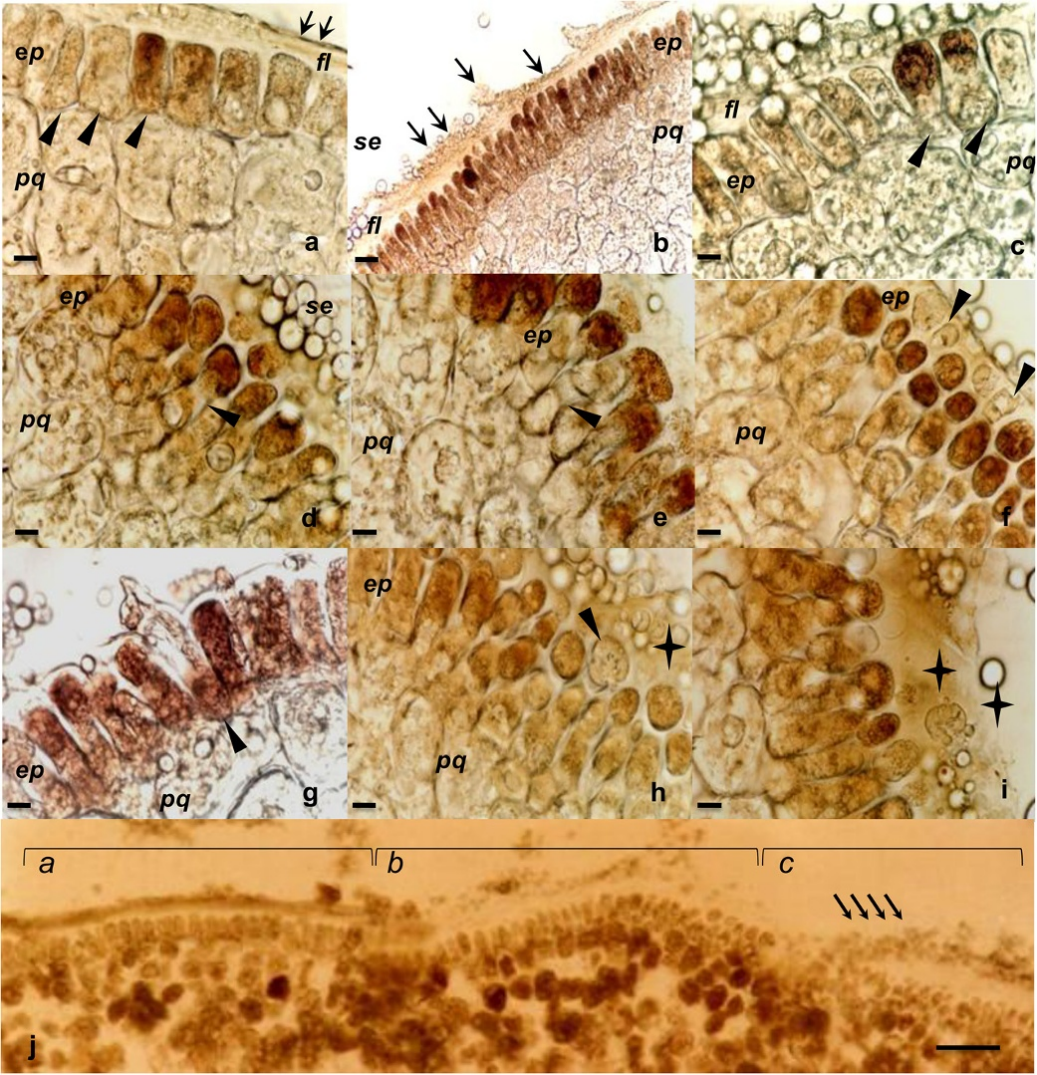
**Changes in epidermal cell morphologies following 24 hrs of imbibition. a**-**j**, *in situ* PODa, AEC + H_2_O_2_; **a**-**b**, view of scutellum epidermis and parenchyma; **c**-**i**, transformation of epidermis, presence of PODa in apoplast, and regional alterations of FL; **j**, area of scutellum where 3 regions are observed, showing asynchrony of transformation process for epidermis and FL; the section was incubated with AEC without H_2_O_2_, showing sites of production of endogenous peroxides. Abbreviations: *ep*, epidermal cells; *fl*, fibrous layer; *pc*, parenchyma cells; *se*, central starchy endosperm. Symbols: ▲, epidermal cells in the process of transformation; ↑, structural changes in fibrous layer; ✦, apoplastic PODa. Bar: a, c-i: 50 μm; b: 125 μm; j: 500 μm.

After 24 h of imbibition, the epidermis that was adjacent to the FL showed monostratified areas (Figure 3a), similar to that which was reported by (Enríquez-Arredondo et al. 2005). Some of the epidermal cells were cuboid, while others showed expanded basal poles (Figure 3a). In other regions, the epidermal cells had lost their regular monostratified arrangements (Figure 3b) and showed PODa *in situ* with increasing staining intensities towards their apical regions (Figure 3c, 3d and 3e; Additional file 1a). Some cells developed elongated finger-like extensions (Figure 3b, 3g), while others had perforated cell walls (Figure 3f), allowing for the extrusion of the cellular contents into the apoplastic space between the epidermis and the FL (Figure 3g and 3h), which turned red-brown as a result of PODa (Figure 3i). These findings were confirmed by the activity that was detected by aminoethylcarbazole (AEC) or guaiacol in the germinator (Additional file 1b).

The aforementioned anatomical changes are asynchronous over the surface of the scutellum, occurring three regions that possess different degrees of modifications of the epidermis and fibrous layer. For example, the monolayered (Figure 3ja) region passes through the epidermis, which extrudes its contents (Figure 3jb) to a region with extensive changes in the FL (Figure 3jc). Later, at 36 h imbibition, the epidermal cells becomes papillate cells (Additional file 1c), which have been reported by Szcziparev (2006) to acquire the characteristics of an epithelium.

### Quantification of peroxidase activity in scutellum

**Figure 4:**
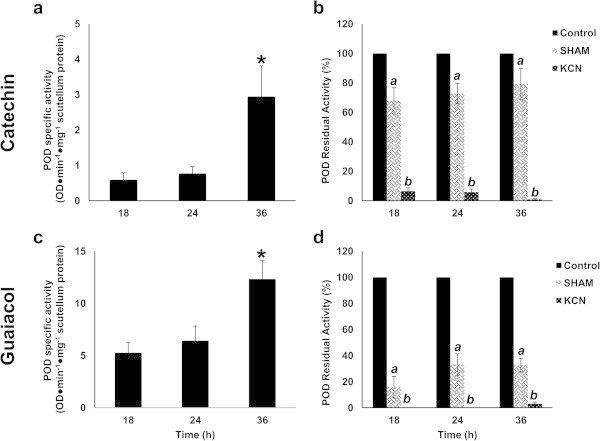
**Peroxidase activity in maize scutella. a**, activity detected with catechin + H_2_O_2_; **b**, inhibiting effects of KCN and SHAM in presence of catechin + H_2_O_2_; **c**, activity detected with guaiacol + H_2_O_2_; **d**, inhibiting effects of KCN and SHAM in presence of guaiacol + H_2_O_2_. Results are shown as means ± standard deviations (SD), n = 6. An ANOVA test and Tukey-Kramer Multiple-Comparison Test were performed with p < 0.05. Symbols: data groups with different letters imply significant differences; *, statistically significant group; *a*, significant effect of SHAM with respect to control; *b*, significant effect of KCN with respect to control.

Because of the correlation between the alterations in the tissues on the scutellum surface and the location of PODa, it was of interest to quantify the PODa in the scutellum between 18 and 36 h of imbibition. The results indicated the presence of 30% and 22% increased PODa levels when catechin and guaiacol were the substrates between 18 and 24 h, and more dramatic increases (282% and 92%, respectively) between 24 and 36 h (Figure 4a and 4c). The differential proportion of activity between catechin and guaiacol amounted to 8.9-fold at 18 h, 8.3-fold at 24 h, and only 4.1-fold at 36 h, indicating that increases in PODa occur that are dependent upon the substrate and that different peroxidase isoforms may be preferred during germination. Using peroxidase inhibitors, it was observed that, between 18 and 36 h, KCN induced between 93% and 99% inhibition when catechin was the substrate and between 96% and 99% inhibition when guaiacol was the substrate. The addition of salicylhydroxamic acid (SHAM) during the same period induced between 20% and 30% inhibition when catechin was the substrate and between 67% and 84% inhibition when guaiacol was the substrate (Figure 4b and 4d). These results confirm the activity of phenol peroxidase in the scutellum.

### Quantification of extruded reduction power

**Figure 5:**
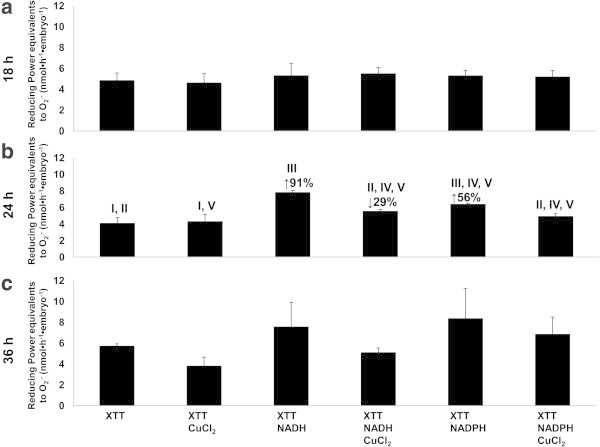
**Superoxide equivalents extruded by scutellar epidermis.** Reduction of XTT by substances produced from surface of scutellum in presence of one of the following conditions: CuCl_2_, NADH, NADPH, NADH + CuCl_2_, or NADPH + CuCl_2_; **a**, after 18 h; **b**, after 24 h; **c**, after 36 h of imbibition. Results are shown as means ± SD of n = 3. ANOVA and Tukey-Kramer Multiple-Comparison Tests were performed with p < 0.05. Symbols: data groups with different numbers imply significant differences; ↑%, represents percent increase in reduction capacity; ↓%, represents percent decrease in reduction capacity.

Superoxide has been reported to reduce XTT (Liszkay et al. 2004) and was observed to diffuse from the surface of the scutellum (Figure 5a-c). The extruded component exhibited similar capacities for basal reduction after 18, 24, and 36 h of imbibition (Figure 5a-c; first column). When 200 μM NADH, 200 μM NADPH, 50 μM of CuCl_2_, NADH + CuCl_2_, or NADPH + CuCl_2_ were added, the reduction capacity that were detected in 18 and 36 h of imbibition were not statistically significant (Figure 5a and 5c). However, in embryos after 24 h of imbibition, the reduction power following the addition of 200 μM NADH or NADPH to the medium increased by 91% and 56%, respectively. The addition of 50 μM CuCl_2_, which has been reported to be a scavenger of O_2_^.-^(Liszkay et al. 2004), did not lead to changes in the basal reduction for XTT *in vivo*. However, the addition of CuCl_2_ in the presence of NADH resulted in a significantly decreased reduction capacity of 29%, and a diminution of 24% was observed with NADPH (Figure 5b), indicating the presence of O_2_^.-^ among the compounds that were extruded from the surface of the scutellum.

**Figure 6:**
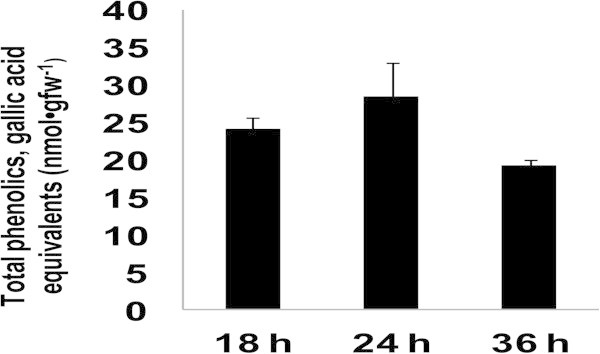
**Scutellum extruded compounds capable of reduction of FeCl**_**3**_-**K**_**3**_ **Fe** **(CN)**_**6**_ **.** Amount of reduced phenols that interact with FeCl_3_-K_3_Fe(CN)_6_. Results are shown as means ± SD of n = 3. ANOVA and Tukey-Kramer Multiple-Comparison Tests were performed with p < 0.05.

Because compounds with reducing capacities were extruded from the scutellum to the apoplast, other components were assayed *in vitro* to determine their interactions with XTT (Table 1). At concentrations within the mM range, the highest reduction capacity was observed with catechin, the lowest with gallic acid, and intermediate levels were detected with ascorbic acid. At concentrations in the μM range, CuCl_2_ showed the same reduction power as gallic acid. The capacity for NADH to reduce XTT was similar to that of CuCl_2_ at equimolar conditions while that of NADPH was slightly lower. These results indicate that the compounds that are extruded from the scutellum (Figure 5, first column) may potentially include other types of reducing factors. It has been reported that the scutellum surface adjacent to the endosperm can reduce ferricyanide (Szcziparev 2006), which had been previously reported for the *in situ* localization of phenols (Price and Butler 1977). For this reason, the FeCl_3_-K_3_Fe(CN)_6_ reduction power was measured in the scutellum extrudates following 18, 24, and 36 h of imbibition, in which the amount of extruded phenols did not significantly change between measurements, although there was a slight increase after 24 h of imbibition of 17% or 47% when compared with the measurements that were obtained after 18 or 36 h of imbibition (Figure 6). These results indicate that reduced phenols in the apoplast can be found within a timeframe that is strictly regulated and coincides with the maintenance of basal values of XTT reduction (Figure 5, first column).

**Table 1:** **Compounds effects in reduction of XTT** ***in vitro***

Substance	XTT reduction equivalents (nmol) ^a^
CuCI_2_ (50 uM)^b^	1.71 ± 0.28
Gallic acid (7.8 mM)^c^	1.72 ± 0.87
NADPH (200 uM)^d^	4.36 ± 1.26
NADH (200 uM)^d^	6.79 ± 0.39
Ascorbic acid (50 mM)^c^	26.45 ± 0.22
Catechin (5 mM)^e^	48.04 ± 5.75

^a^the equivalent reducers were determined using the molar extinction coefficient of XTT: ϵXTT = 2.16 X 1O^4^mol^-1^ cm^-1^ (Sutherland and Learmonth 1997); ^b^concentration reported as O_2_-scavenger (Liszkay et al. 2004); ^c^concentrations of phenols per embryo, the phenols are detached from the scutellar surface in maize embryos with 24 hours of imbibition; ^d^concentrations reported by Frahry and Schopfer (2001); ^e^concentration calculated from constant rates of ROS scavenging.

### Effects of storage on peroxidase activity, vigor and autofluorescence of endogenous compounds

**Figure 7:**
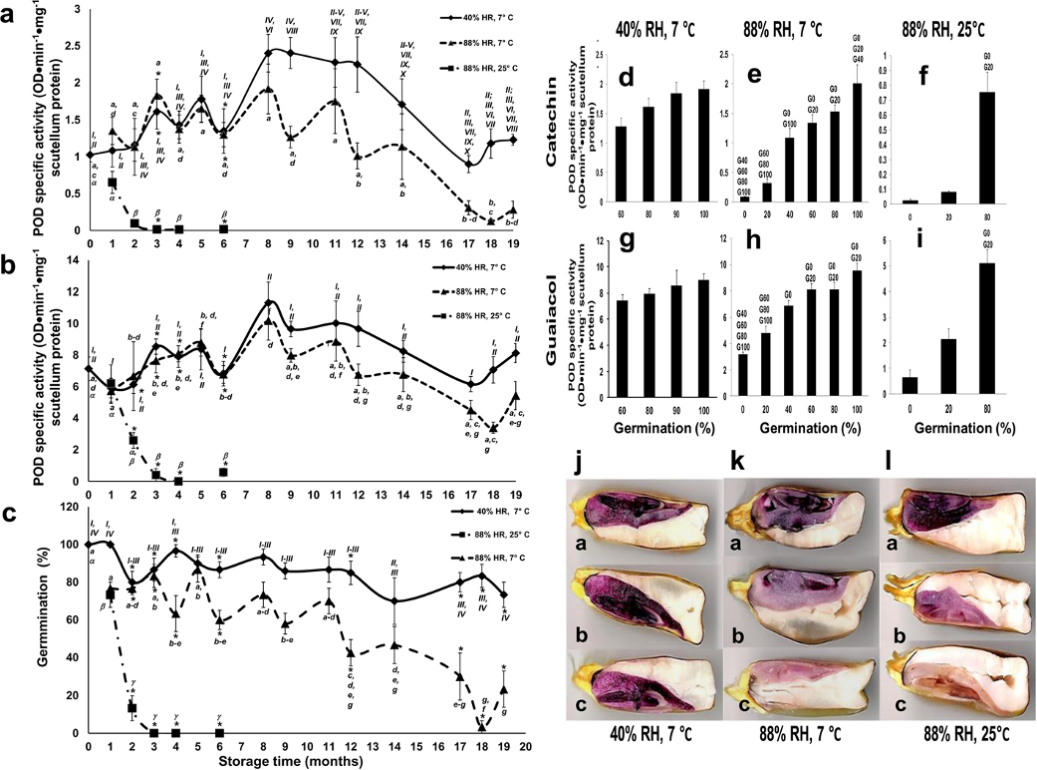
**Quantification of peroxidase activity in scutella of grains kept under controlled storage**; **germination and vigor assay.** PODa is shown, with: **a**, catechin + H_2_O_2_, and **b**, guaiacol + H_2_O_2_ in extracts of scutella from maize grains that were stored up to 19 months. Activities were quantified after 24 h of imbibition; **c,** germination after 24 h of imbibition; correlation between germination and PODa, **d**-**f** quantified with catechin + H_2_O_2_, **g-i** quantified with guaiacol; **j**-**l**, vigor assays with MTT, which was applied to grains at end of storage time. Results are shown as means ± SD with up to 12 independent assays. ANOVA followed by a Tukey-Kramer Multiple-Comparison Tests were performed with p < 0.05. Symbols: data groups with different letters or numbers from the same storage conditions imply significant differences; and *, statistically significant differences comparing different treatments with same storage time.

In the scutellum after 24 h of imbibition, the PODa levels fluctuated around a constant value for samples that had been stored between 0 to 6 months at 40% and 88% RH and 7°C with high germination rates. For samples that had been stored at 88% RH and 25°C, after 24 h of imbibition, the PODa levels and germination rates decreased and reached zero in the third month (Figure 7a, 7b, and 7c). 

In the SG between 8 and 12 months at 40% RH and 7°C, the scutellum after 24 h of imbibition showed significantly increased PODa levels compared with the initial values, with maximum activity levels being detected between 8 and 9 months with catechin (Figure 7a) and at 8 months with guaiacol (Figure 7b). These activity levels remained elevated until 12 months. Unlike the SG at 88% RH and 7°C, the PODa with catechin showed similar fluctuations as those which were observed between 3 to 6 months (Figure 7a). The PODa increased at 8 months with guaiacol and then declined at 12 to 13 months to similar levels as those that were detected at the beginning of the experiment (Figure 7b). The SG at 40% RH and 7°C maintained its germination rates at a mean value of 90% until 12 months, while the embryos that had been extracted from storage at 88% RH and 7°C maintained 78.8% germination rates during the first 3 months and fluctuated downward until 12 months, when they reached a significantly lower germination rate of 42% (Figure 7c). 

After 12 months of storage at 40% RH and 7°C, the PODa in the scutella after 24 h of imbibition decreased, reaching its baseline value at 17 months with catechin or guaiacol. This activity is significant with respect to the maximum values that were obtained but is similar to the PODa that was observed at baseline (Figure 7a and 7b). For the same frame time, in SG at 88% RH and 7°C, the PODa decreased below initial levels and reached a minimum value between 17 to 19 months (Figure 7a and 7b). During this time frame, the germination rate for SG at 40% RH and 7°C was similar to that which was found at the start of the study. The germination rate of samples that had been stored at 88% RH and 7°C decreased below 30% with a minimum value of 3.3%, which was observed at 18 months (Figure 7c). 

When storage was extended to 22 months, the SG at 40% RH and 7°C showed high (Figure 7jc) to medium vigor (Figure 7ja,b), whereas that at 88% RH and 7 or 25°C showed low vigor (Figure 7ka; 7la) and in some cases, grains were ungerminable (Figure 7kb, 7kc, 7lb, 7lc). 

The storage conditions at 88% RH and 25°C or 7°C caused a fast or slow deterioration, respectively. Accordingly, the loss of grain viability with a concomitant diminution in PODa below the baseline, which was recorded at the beginning of the assay in embryos following 24 h of imbibition, was detected. Moreover, in SG at 40% RH and 7°C, a high vigor and germination rate was found in accordance with the baseline value or higher levels of PODa at 24 h of imbibition (Figure 7d-i).

**Figure 8:**
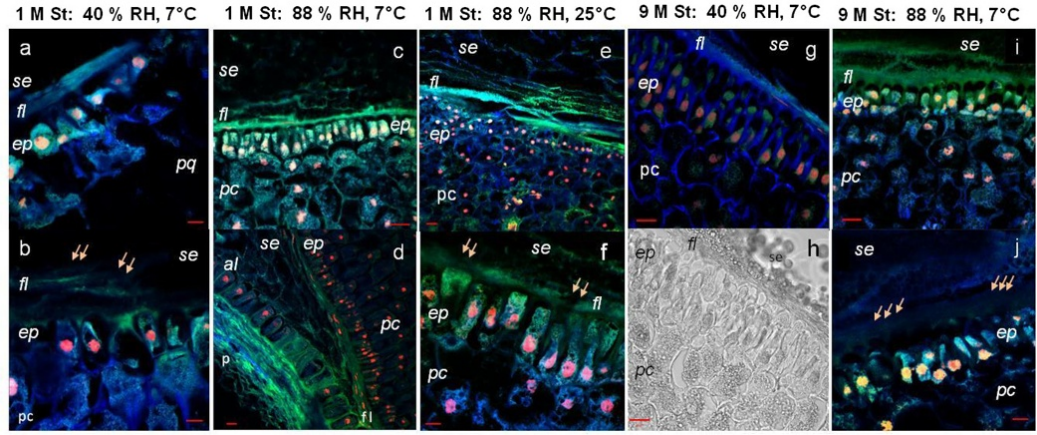
**Autofluorescence of scutella after 24 h of imbibition from grains stored under controlled conditions. a**-**f**, 1 month of storage; **g**-**j**, 9 months of storage; where **a**, **b**, **g**, and **h** at 40% RH and 7°C; **c**, **d**, **i** and **j** at 88% RH and 7°C; **e** and **f** at 88% RH and 25°C. Abbreviations: *al*, aleurone layer; *ep*, epidermis; *fl*, fibrous layer; *p*, pericarp; *pc*, parenchyma cells; *se*, central starchy endosperm. Symbols: →, modification of emission by the FL. Bars: **a**, **b**, and **f**: 10 μm; **c**-**e** and **g**-**j**: 20 μm.

Because PODa levels increased at 9 months of storage in comparison with levels that were observed at the beginning of the assay and germination rates remained high, a comparative anatomical study was performed between embryos (at 24 h of imbibition) from the SG after 1 and 9 months. The results showed that the anatomy of the scutellum epidermis corresponded with monostratified and uninucleate cell layer, which were independent of storage conditions (Figure 3a-e and Figure 8). The scutellum and FL of the embryos of the dry grain (Additional file 2) showed autofluorescence in three parts of the spectrum: red (F_626–635__nm_) > green (F_512 nm_) > blue (F_460 nm_). After 24 h of imbibition, the scutella from the SG at 40% RH and 7°C for 1 and 9 months had lower proportion of green *vs* blue emissions compared with those that were unimbibed (Additional file 2). The FL was observed to fluoresce blue, varying in appearance between a fibrous (Figure 8a) and loose consistency (Figure 8g), and in some specific areas, autofluorescence disappeared (Figure 8b), although the scutellum remained structured (Figure 8h). In the scutellar epidermis and the FL from the SG after 1 month at 88% RH and 7°C from 24 h of imbibition, green fluorescence predominated, and blue fluorescence was observed in the remainder of the scutellum (Figure 8c). In the basal area of the scutellum, where the epidermis, SE, and aleurone layer meet (Figure 8d), the FL maintained its green fluorescence. After 9 months, the parenchyma of the scutellum maintained its emission patterns, whereas the FL, which had a loose structure, and the epidermis showed predominately green (Figure 8i) or blue (Figure 8j) fluorescence. After 1 month, the scutella of the SG at 88% RH and 25°C had an epidermis that displayed blue autofluorescence (Figure 8e). Additionally, the apical section of the epidermis emitted green autofluorescence, and in the basal portion of the cell, blue fluorescence predominated (Figure 8f). The FL primarily fluoresced green, either continuously or in small sections, and the subjacent parenchymal cells fluoresced blue (Figure 8e and 8f).

## Discussion

The presence of an FL limiting the SE, which has been reported by Enríquez-Arredondo et al. (2005), has been confirmed in the present study using maize grains of Chalco. It was observed that the FL forms a continuous layer that envelopes the embryo from the basal to the apical region. In maize, the FL consists of compacted cellular walls that originate from the peripheral strata of the SE, which is in contact with the scutellum (Bourdu and Bousser 1991) and is homologous to the structure that is found in other cereals, such as rice (Bechtel and Pomeranz 1977; Okamoto et al. 1982), wheat (Swift and O'brien 1972), and barley (MacGregor and Matsuo 1982; MacGregor and Dushnicky 1989).

This study adds to existing reports describing the chemical composition of the FL, which includes collapsed walls that contain lipids, phenols, and a β-glucan of the callose type. The presence of phenols is in accordance with the distribution of compounds that surround the scutellum, which was previously described but not defined by Sen et al. (1994) and Bergvinson and García-Lara (2004). The callose in the cell walls is known to contribute to the hermetic sealing of the tissues where it is deposited (Parre and Geitmann 2005; Tiwari 1982) and to provide a matrix for the association of other components of the cell wall (Eschrich 1975), including phenols, which participate in the assembly of polysaccharides with structural proteins (Fry et al. 2000; Saulnier and Thibault 1999). Some of them may form suberin or cutin by chemical bonds that are formed with lipids (Kolattukudy 1981), which occurs in the embryonic sac or in the cuticle-like layer that is found in the soybean (Chamberlin et al. 1993), thus contributing to the generation of semi-permeable structures, such as the envelope of the perisperm-endosperm that is found in muskmelon and cucumber seeds (Amritphale et al. 2010; Ramakrishna and Amritphale 2005; Yim and Bradford 1998). Crushed cell walls in the FL with deposits of β-glucan of the callose type may form a mesh with pores in which lipids and phenols could be inserted, contributing to the function of the FL as a diffusion barrier (Figure 1 and 2). This barrier generates two separate compartments, and in this way, the embryo remains isolated in the dry grain.

The independence that is maintained by the two compartments when the FL is intact may benefit the embryo because it enables the compartmentalization of signals and metabolic processes that are produced during germination. This compartmentalization prevents the diffusion of signals and other components to the rest of the grain and maintains the optimum concentrations of the metabolites that are necessary during this stage. During early post-germination, an increase in the nutrient demand by the growing embryo axis leads to a decrease in the reserves of the scutellum. Therefore, it is indispensable for the embryo to transform the FL, enabling the diffusion of amylolytic enzymes from the scutellum to the endosperm and of signals from the embryo to the aleurone, inducing amylase and protease synthesis (among other enzymes), allowing for the absorption of hydrolyzed reserves from the SE by the scutellar epithelium (Okamoto et al. 1982; Szcziparev 2006).

The transformation of the FL is performed in small areas at 24 h of imbibition, correlating with structural and functional modifications of the epidermal cells. The isolation between the embryo and endosperm compartments is eliminated, enabling the diffusion of substances between the SE and epidermal cells from the scutellum. For this process, some alterations occur, in which the epidermal cells become elongated, and the basal pole is expanded, acquiring finger-like extensions and then some perforate the cell walls (Figure 3j). This is accompanied by the extrusion of components to the apoplast, which have the capacity to reduce XTT, indicating that the scutellum generates O_2_^.-^ (Figure 5b) and release phenols (Figure 6) and likely other compounds (Table 1). In addition to O_2_^.-^ and phenols, PODa was detected in the apoplast (Figure 3), which uses phenols, such as guaiacol (García-Lara et al. 2007) and catechin, as substrates and can be inhibited by specific inhibitors (Figure 4; Liszkay et al. 2004). This in turn contributes to the modification of the FL (Figure 3j). After 36 h of imbibition, these cells are associated with the absorption of nutrients that originate in the endosperm, corresponding with the role of the functional epithelium as described by Szcziparev (2006).

PODa may possess dual functions because it acts as an antioxidant during stress conditions but can also act as a pro-oxidant under other conditions. In the apoplastic microenvironments of the scutellum, an oxidative burst occurred at 24 h of imbibition (Figure 5b). During this time, O_2_^.-^ were generated via an oxidative cycle of the enzyme (Liszkay et al. 2003). It has often been suggested that in addition to NAD(P)H oxidases, peroxidases may also serve as sources for apoplastic O_2_^.-^ production *in vivo* (Bolwell et al. 2002; Minibayeva et al. 2009). NAD(P)H oxidase or a portion of its activity can be attributed to the RBOH-type flavoenzyme, which is the plant equivalent of the gp91^phox^ subunit of NADPH oxidase of mammalian phagocytes (Sagi and Fluhr 2006). Peroxidases exhibit O_2_^.-^-producing activities only if they are exogenously supplied with an electron donor, such as NAD(P)H, at high concentrations (Chen and Schopfer 1999). However, all attempts to identify a genuine electron donor for the peroxidases *in vivo* were unsuccessful (Heyno et al. 2011). Subsequently, the activation of the hydroxylic cycle of PODa may lead to the consumption of the O_2_^.-^ or H_2_O_2_ generating °OH (Liszkay et al. 2003), in which case apoplastic hydroxyl radical generation depends mainly upon peroxidases that are localized in the cell walls or even membranes (Heyno et al. 2011; Mika and Lüthje 2003). This radical may modify components of the cell wall. The interaction between the ROS that are generated by PODa and the components of the FL in maize would alter this structure, similarly to the previously proposed model of the root elongation by Schopfer (2001), that suggesting when the structure of the FL is modified, its resistance to diffusion is eliminated.

In support of the previous discussion, it has been reported that *in vitro* and at alkaline pH levels, some flavonoids, such as catechin, can initiate an autooxidative cycle that could produce O_2_^.-^ and H_2_O_2_ (Mochizuki et al. 2002). Moreover, it has been reported that polyphenols, which normally act as antioxidants, can be converted to pro-oxidants by hydrolysis. The pro-oxidant activities of phenols depends of their chelating behaviors, metal reducing properties, and O_2_^.-^ reducing capacities, which are related to the redox potentials of the oxidized species and the lifetimes of the phenoxyl radicals (Sakihama et al. 2002).

However, in a broader context, PODa might function as an antioxidant when the grain is exposed to stress conditions. During seed storage, a loss of vigor and germination abilities occurs. The degree of seed degeneration depends upon specific protection mechanisms, including the activities of antioxidant enzymes, such as superoxide dismutase, catalase, and peroxidase (Balešević-Tubić et al. 2005, 2010; Chauhan et al. 2011; Goel and Sheoran, 2003). PODa acts as an antioxidant because it displays a peroxidative cycle, during which H_2_O_2_ is consumed by the oxidation of various substrates, such as phenols (Liszkay et al. 2003). It has been demonstrated that flavonoids and hydroxinamic acids are capable of scavenging H_2_O_2_ by acting as electron donors for guaiacol peroxidases (Sakihama et al. 2000). When phenols act as antioxidants, they are univalently oxidized to their respective fenoxil radicals, either by direct radical scavenging mechanisms or by an enzymatic pathway (Kagan and Tyurina 1998). Particularly, polyphenols, as opposed to monophenols, act as antioxidants (Sakihama et al. 2002). For this reason, the antioxidant/pro-oxidant capacities of phenols depend upon microenvironmental factors, such as pH, solubility, chelating behaviors, and metal-reducing potentials (Decker 1997).

The decreased PODa levels that have been observed in various seeds is associated with losses of viability and germination capacities during storage (Balešević-Tubić et al. 2005, 2011; Bao et al. 2011; Chauhan et al. 2011; Goel and Sheoran 2003; Scialabba et al. 2002). In stored maize, increased temperatures in conditions of high humidity (88% RH at 25°C) lead to rapid deterioration after 3 months with decreased germination rates and abrupt decreases in scutellum PODa levels (Figure 7c). This deterioration is in accordance with previous reports of maize grains that were stored at 75% RH and 35°C (Bernal-Lugo and Leopold 1992). In the first 6 months of storage at 88% RH and 7°C, POD and germination rates fluctuated over baseline values, with considerable losses of germination and PODa being observed after 12 months. The FL and scutellar epidermis from these SG exhibited autofluorescence with increased green compared with blue emissions (Figure 8c-f, 8i, and 8j), which are qualitatively similar to those from the scutella of dry grains (Additional file 2), indicating that those embryos did not experience or complete their alterations of the components of the scutellum epidermis and the associated FL at 24 h of imbibition, revealing that the grains presented with low vigor to ungerminability at 22 months. Storage at high humidity levels (88% RH) provokes a cumulative oxidative stress that exceeds the acceptable window (Bailly et al. 2008), resulting in the deterioration and concomitant loss of vigor of the seed. This process is fast at high temperatures (25°C) and slower at low temperatures (7°C), leading to a lack of capacity for maintaining basal antioxidant levels, such as those of PODa in the scutellum, during germination. The relationship between the decreased PODa in the scutellum and loss of viability that was observed in the maize grain has also been observed in sunflower seeds and soybean (Balešević-Tubić et al. 2005, 2011).

The SG at 40% RH and 7°C maintained a high germination capacity, while the PODa in the scutellum after 24 h of imbibition increased from 6 to 12 months of storage. These results imply that the embryos, starting at 6 months of storage, are subjected to stress levels that are likely lower than those that are found in the SG at 88% RH and 7°C, given that the deterioration is less severe and vigor is maintained at high-medium levels for a longer time period. Maintaining high-medium vigor is sufficient to induce increases in protective mechanisms during imbibition, thus keeping the ROS concentrations within an acceptable range. PODa protects the scutellum by counteracting the stress that is provoked during storage and germination, helping to maintain oxidant conditions within an acceptable window (Bailly et al. 2008), which enables the embryonic root to grow vigorously and establish the seedling. High vigor and germination in association with activity levels exceeding the baseline allows for the proportion of green emission to decrease in the scutellum after 24 h of imbibition, permitting the observation of blue fluorescence in the epidermis and FL or the disappearance of autofluorescence in some areas. (Figure 3, Figure 8a, 8b, 8g, 8h). The differences in the proportions of blue and green fluorescence under the conditions that were studied indicate that there may be alterations in certain emission properties (for example, by effect of pH or ionic strength) or the proportions of components of integral elements of the FL and epidermis. These include phenols, such as cinnamic acids and catechin, which fluoresce blue, or anthocyanins, quercitrine, quercitin, and kaempferol, which fluoresce mainly green with a basal shoulder which emits in the blue region (Harborne 1973; Lang et al. 1991), correlating with alterations in microenvironmental conditions and changes in the anatomies of the scutellum and FL. These results imply that a low temperature and low relative humidity level enables the conservation of grain vigor and viability for longer time periods when compared with storage at higher temperatures and humidity levels, confirming the observations that were reported by Eskandari (2012). The maintenance of high vigor enables the increase in scutellum PODa levels as a protective mechanism against the stress that occurs during storage and germination.

## Conclusion

These findings suggest that, based on its histochemical composition, the FL represents a physical barrier that prevents free flow between the scutellum and endosperm. The FL keeps the isolation and independence of the two compartments in the dry grain, this compartmentalization is beneficial to the embryo because it prevents resource diffusion to the endosperm. Therefore, the concentrations of metabolites are maintained at optimum levels. Nutritional requirements increase when the embryonic root begins to elongate. Accordingly, the epidermis of the scutellum is modified and peroxidase activity is heightened in the apoplast along with the emergence of higher levels of superoxide and maybe other radicals. These elements react with compounds of cell wall, enabling the transformation of the FL, thus allowing for free flow between the chambers. The PODa, due to its dual function, may participate in the process of FL modification in small areas or microenvironments by generating ROS, which is a process that must be tightly regulated. Outside of these microenvironments, the PODa of the scutellum acts broadly as an antioxidant enzyme for the fine control of ROS levels to avoid damage, counteracting the oxidative stress that occurs during storage and germination and conserving the integrity and functionality of the scutellum. This contributes to the high vigor of the embryo and its capacity to establish the seedling. Therefore, the PODa in the scutellum and proportion of F_green_*vs* F_blue_ emission after 24 h of imbibition may be used as indicators of the degree of deterioration of the SG.

## Materials and methods

### Biological materials

The source of grains was creole maize cultivated in Valle de Chalco, Mexico. For the experiment, up to 12 replicas by assay with ten grains were used, as follow: 1) whole grains were used to determine the diffusion capacity through the FL; 2) whole grains were imbibed for 12 h for viability tests with tetrazolium. 3) embryos were manually dissected from the ripe grains to determine the FL compositions, and 4) embryos were dissected, superficially disinfected with 3% NaClO for 5 min, and washed 3 times with sterile distilled water. They were then incubated in moist germinators in the dark at 25°C for the following time periods: a) 18, 24, or 36 h time points were used to quantify PODa in the scutellum, the compounds that were extruded into the apoplast near the scutellum with the capacities to reduce 2,3-bis(2-methoxy-4-nitro-5-sulfophenyl)-2H-tetrazolium-5-carboxanilide inner salt (XTT), and the liberation of phenols using the Prussian Blue technique; b) 24 or 36 h time points were used for the *in situ* localization of PODa and its anatomical correlations; and c) a 24 h time point was used for grains at various stages that originated from controlled storage to determine the germination capacity and PODa content of the scutellum and to detect the autofluorescence of endogenous compounds that are associated with its anatomy.

### Histochemical detection of components of fibrous layer

#### Fixation, infiltration, and cutting

Embryos from dry grains were fixed in 4% paraformaldehyde (pH 6.9), infiltrated in 300 mM saccharose, and processed as described by Enríquez-Arredondo et al. (2005).

#### Detection of lipids

Histological sections were hydrated for 10 min and treated using 1 of 2 methods: a) for the detection of neutral lipids and esters, sections were immersed in 60% ethanol for 10 min and stained with a 0.5% Oil Red O solution in propanol:water (6:4, v:v) for 15 min, and the excess stain was washed with distilled water (Krishnamurty 1988; Pearse 1968); and b) for the detection of lipid components, the sections were stained for 15 min with a 0.09% Sudan IV solution in glycerol:ethanol (1:1, v:v), and the excess stain was washed off with distilled water (Ruzin 1999). The preparations were mounted in an aqueous medium and observed with Nomarski differential interference contrast microscopy using an Axoskop Zeiss microscope.

#### Detection of phenolic compounds

For the *in situ* detection of phenols, histological sections were treated for 10 min with ethyl acetate and then incubated for the same length of time in 0.5% diphenylborinic acid that was dissolved in ethyl acetate (Reich et al. 2006). Excess reactivity was eliminated by absorption, and the detection of phenolic compounds was performed with an epifluorescence Axoskop Zeiss microscope, using an excitation wavelength of 365 nm and an emission filter for fluorescein.

#### Detection of 1,3-β-glucan (callose)

Aniline blue interacts with glucose residuals containing β-1,3 bonds to form fluorescent compounds and is used to detect polysaccharides, such as callose (Smith and McCully 1978). The sections were hydrated for 10 min and treated with 0.01% aniline blue that was dissolved in 0.1 M phosphate buffer (pH 9) for 15 min. Excess stain was eliminated by absorption. A 365 nm wavelength was used for the excitation of the aniline-β-1,3 polysaccharide complex, and its emissions were observed in the blue region (Ruzin 1999) using an epifluorescence Axoskop Zeiss microscope.

#### *In situ*localization of peroxidase activity

For the determination of the *in situ* localization of PODa in the scutellum, embryos that had been imbibed for 24 or 36 h were incubated for 30 min at 25°C in 50 mM sodium acetate buffer (pH 4.5) with 0.22% H_2_O_2_ and 0.05% 3-amino-9-ethylcarbazole (AEC) that was dissolved in N,N-dimethylformamide (Graham et al. 1965), followed by fixation, infiltration, and cutting as previously described. Embryos that had been incubated in the same solution without H_2_O_2_ were used as controls. The histological sections were observed with Nomarski differential interference contrast microscopy using an Axoskop Zeiss microscope.

### Diffusion and permeability assays

#### Grain perforation and capacity for dye diffusion through fibrous layer

Dry grains were perforated either on the embryo or endosperm side 3 times using a dissecting needle. To evaluate the diffusion between the two compartments, an I_2_-KI aqueous solution, which was prepared according to (Landry and Smyth 1988), was used to stain the starch in the endosperm and scutellum, and a 0.5% Brilliant Green solution (Merck 1310) that was dissolved in 95% ethanol was used to stain the cytoplasm and cell walls green. The perforated grains were incubated at 4°C for 24 h in the aqueous solution or 48 h in the ethanol solution. The excess stain was removed by washing exhaustively with distilled water to prevent the contamination of the cutting knife when the grains were cut longitudinally, and stained areas were observed to establish the diffusion between the two compartments. Stain diffusion in unperforated or perforated grains from the embryo to the endosperm were also evaluated.

#### Extraction of components from fibrous layer and effects on permeability

Grains that had been perforated through the embryo or endosperm were incubated for 4 days in acetone, chloroform, or acetone:chloroform (1:1, v:v) at room temperature. After 11 days of solvent evaporation, the diffusion was established as previously described.

### Quantification of peroxidase activity in scutellum

In embryos that had been imbibed for 18, 24, or 36 h, the scutella were dissected from the embryonic axes on an ice bed and weighed. The embryonic axes were discarded to eliminate the high PODa levels that were present in the areas of embryonic root growth (Liszkay et al. 2004). The scutella were homogenized in 3 ml of 100 mM phosphate buffer (pH 6.8) and centrifuged at 10,000 x g for 30 min at 4°C. The supernatants, which were used as raw extracts, were maintained at 4°C until analyzed (García-Lara et al. 2007). The protein concentrations were determined using the method described by Lowry et al. (1951) with bovine serum albumin as the standard. PODa was determined using the following 2 methods: a) to begin the reaction, 4 μl of 3% H_2_O_2_ were added to the reaction mix, consisting of 440 μl of 50 mM phosphate buffer (pH 6.8), 20 μl of enzyme extract, and 440 μl of 20 mM (+)-catechin, and the absorbance at 475 nm wavelength was recorded every minute for 5 min; and b) 870 μl of 50 mM phosphate buffer (pH 6.8), 10 μl of enzyme extract, 10 μl of 1 M guaiacol, and 9 μl of 3% H_2_O_2_ were added to begin the reaction, and the absorbance at 475 nm was recorded every 20 s for 2 min. PODa inhibitors, which included either 10 μl of 0.3 M salicylhydroxamic acid (SHAM) or 10 μl of 0.1 M KCN (Liszkay et al. 2004), were added to the solutions that are described above, and the quantification of activity levels was performed as described. For the spectrophotometric assessments, a Perkin Elmer UV/VIS Spectrometer Lambda 2 was used.

### Reducing capacity of contents extruded from scutellum

#### Quantification of extruded reducing power: XTT reduction by O_2_^.-^ equivalents

The reducing capacities for XTT were determined in embryos that had been imbibed for 18, 24, or 36 h. Briefly, embryos with different imbibition times were placed into a Costar 6-well culture plate with the sides of the scutella that were exposed to the SE facing the bottoms of the wells. The embryos were incubated in 1 ml of a 500 μM XTT solution (Liszkay et al. 2004) in a 10 mM potassium citrate buffer (pH 7.4; Sagi and Fluhr 2001) for 1 h in the dark. The absorbances at 470 nm were then quantified. The blank consisted of 1 ml of the same solution that had not been in contact with the embryos.

To determine the effects of NADPH, NADH, and CuCl_2_, embryos were treated as described above and placed into one of the following solutions: 200 μM NADH, 200 μM NADPH, 50 μM CuCl_2_, 200 μM NADH with 50 μM CuCl_2_ or 200 μM NADPH with 50 μM CuCl_2_ in 10 mM potassium citrate buffer (pH 7.4) with 500 μM XTT (Liszkay et al. 2004). The absorbances of the solutions at 470 nm were then quantified. The blank consisted of 1 ml of the same solution that had not been in contact with the embryos.

#### Solutes with capacities to reduce XTT

To determine the contributions of other compounds towards the reduction of XTT, the following substances were added to 1 ml of 10 mM potassium citrate buffer (pH 7.4) containing 500 μM XTT: 200 μM NADH, or 200 μM NADPH, or 50 mM ascorbic acid, or 5 mM catechin, or 50 μM CuCl_2_, or 7.8 mM gallic acid. The mixtures were incubated at 25°C in the dark, and the absorbances at 470 nm were measured at 5 and 60 min after the reaction began, using the corresponding reduction factor. The equivalent reduction factors were determined by subtracting the final absorbances from the initial absorbances and using the coefficient of molar extinction of XTT (ϵ_XTT_ = 2.16X10^4^ M^-1^ s^-1^) as reported by Sutherland and Learmonth (1997).

#### Phenol release measured by ferricyanide reduction

Embryos were obtained after 18, 24, or 36 h of imbibition. They were deposited into a Costar 6-well culture plate and incubated in 1 ml of distilled water for 10 min, and thus prevented the radicle from coming into contact with the water. Aliquots of 100 μl were retrieved and mixed with 60 μl of 0.1 M FeCl_3_ in 0.1 N HCl. After shaking, 60 μl of 0.008 M K_3_Fe(CN)_6_ in 0.1 N HCl were added, bringing the volume to 1.12 ml in water. The mix was incubated at room temperature for 10 min, absorbances at 720 nm were recorded, and the data were interpolated into a gallic acid standard curve (Price and Butler 1977).

### Effects of storage on peroxidase activity, vigor and emission of endogenous compounds

#### Conditions of controlled storage and quantification of peroxidase activity

Maize grains were stored (SG) in an airtight container under the following controlled conditions: a) 40% relative humidity (RH) at 7°C in a refrigerator with a system for humidity control; b) 7°C in a chamber with a RH of 88%, which was generated using a solution of 10% sodium benzoate (Rockland 1960); and c) 25°C in a chamber with a RH of 88%, which was similar to that described in b. Every 30 days, a sample of grains from each treatment was collected and manually dissected to separate the embryos. The embryos were then disinfected and imbibed. Percent germination was recorded, and a raw enzymatic extract was obtained from the scutella to quantify PODa.

#### Autofluorescence of endogenous compounds

Samples from grains that had been stored in controlled conditions were collected at 1 and 9 months. The embryos were dissected after 24 h of imbibition, fixed in 70% ethanol at room temperature, dehydrated, and infiltrated in paraplast. Sections that were 8 μm thick were mounted onto microscope slides using a gelatin cover. Paraplast was removed, and the sections were hydrated and incubated with propidium iodide to detect the nuclei (Ruzin 1999). They were mounted in aqueous media, and their anatomies were assessed using a Confocal Spectral Carl Zeiss Microscopy System Model LSM 780 NLO. Independent tracks were used, including two for autofluorescence and a third to locate the nuclei with the following parameters: a) excitation: 405 nm, emission: 410 to 516 nm; b) excitation: 488 nm, emission: 490 to 560 nm; and c) excitation: 561 nm, emission: 566 to 685 nm.

#### Vigor assays

To determine the vigor of the SG under controlled conditions, they were collected after 20 to 22 months and imbibed for 12 h. The vigor was determined using the technique that was described by DeVries and Goggi (2006) with slight modifications. The imbibed grains were longitudinally dissected, and one of each of the halves was soaked in water for 10 min. Then, the water was replaced with a 0.1% thiazolyl blue tetrazolium bromide solution (MTT) to stain the grains for 70 min at room temperature. After staining, the MTT solution was drained, and the grains were well rinsed with water. Finally, the stained grains were scanned.

### Statistical analyses

Quantitative results were expressed as the mean and standard deviation from 3 to 12 independent assays. A one-way ANOVA was performed followed by a Tukey-Kramer Multiple-Comparison Test with a significance level of p < 0.05. Analyses were performed with the NCSS and PASS 2000 software.

## Electronic supplementary material

Additional file 1: ***In situ*** **localization of PODa in the scutellum.** Embryos that had been imbibed for 24 h were incubated for 30 min at 25°C in a 50 mM sodium acetate buffer (pH 4.5) with 0.22% H_2_O_2_ and 0.05% 3-amino-9-ethylcarbazole (AEC) dissolved in N,N-dimethylformamide (Graham et al. 1965). Enzymes that were extruded during imbibition of the embryo were detected by reacting the germinator with 0.22% H_2_O_2_ and 0.05% 3-amino-9-ethylcarbazole (AEC) that was dissolved in N,N-dimethylformamide in 50 mM sodium acetate buffer (pH 4.5) for 30 min at 25°C or incubated in a solution with 0.22% of H_2_O_2_ and 10 mM of guaiacol in 50 mM phosphate buffer (pH 6.8) for 15 minutes at 25°C. Both of these reactions were carried out in the dark. For the histochemical sections, the embryos were incubated with AEC and H_2_O_2_, followed by fixation, infiltration, and cutting, and were observed with Nomarski differential interference contrast microscopy using an Axoskop Zeiss microscope. Results. There are high PODa levels at the scutellum surface that is in contact with the SE in addition to the embryonic root region (Additional file 1a). During imbibition, PODa is extruded from the embryo (Additional file 1b), confirming its activity in the apoplast via histological sectioning. A recapitulation of epidermal cell morphology after 24 hours of imbibition and papillate cell after 36 h of imbibition are shown in the Additional file 1c. Peroxidase activity at scutellar surface and extrusion to germinator. a, *in situ* PODa in embryo; b, PODa extruded from embryo to germinator after 24 h of imbibition; c, recapitulation of epidermal cell morphology after 24 h of imbibition and papillate cell after 36 h of imbibition. (TIFF 4 MB)

Additional file 2: **Emission spectrum of the scutellum at 0 h of imbibition.** Embryos of dry grains were fixed in 70% ethanol at room temperature for one day, and then dehydrated, infiltrated, and embedded in paraplast. Sections of 8-μm thickness were obtained and mounted on a microscope slide cover with a gelatin film. The support media were extracted from specimens and mounted in an aqueous medium at pH 7.0, and the emissions of the visible spectrum were recorded following excitation at 405, 458, 488, 514, 561, or 633 nm with a laser from the Carl Zeiss Spectral Confocal Microscopy System Model LSM 780 NLO. Result. The emission spectrum of the scutellum at 0 h of imbibition showed 3 emission peaks: blue at 460 nm; green at 512 nm; and red between 626–635 nm. Slight emissions at 617 and 643 nm were also observed. Each box contains the emission wavelength and color corresponding to the visible spectrum. Bar: 20 μm. (TIFF 2 MB)

## Abbreviations

(AEC): Aminoethylcarbazole
(PODa): Peroxidase activity
(SE): Central starchy endosperm
(ROS): Reactive oxygen species
(FL): Fibrous layer
(RH): Relative humidity
(MTT): Thiazolyl blue tetrazolium bromide
(SG): Stored grain
(SHAM): Salicylhydroxamic acid
(XTT): 2,3-bis(2-methoxy-4-nitro-5-sulfophenyl)-2H-tetrazolium-5-carboxanilide inner salt.
