# Supplementary figures and images for: Peroxidase activity in scutella of maize in association with anatomical changes during germination and grain storage
Source: Springerplus. 2014 Aug 2;3:399. doi: 10.1186/2193-1801-3-399 (PMC4128954; doi:10.1186/2193-1801-3-399)

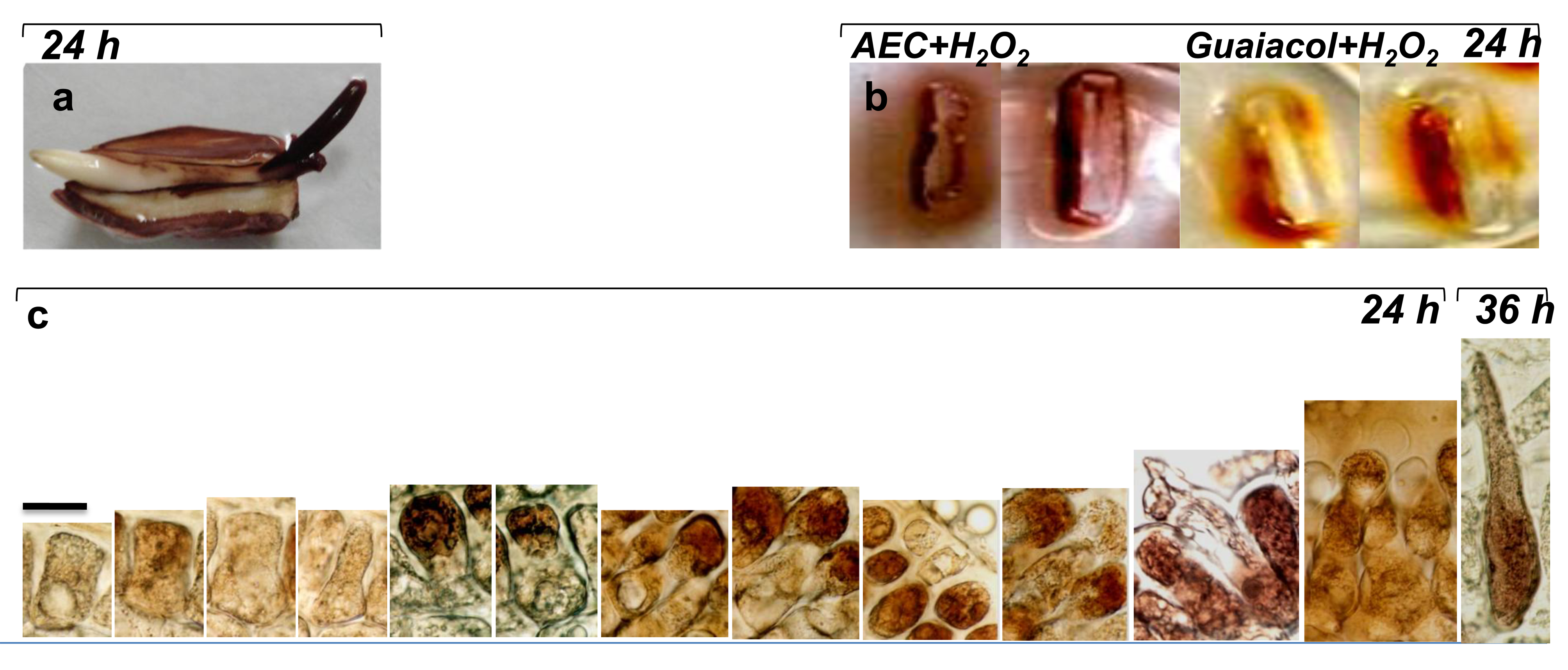

Supplement: Supplementary file 1 — Additional file 1: In situ localization of PODa in the scutellum. Embryos that had been imbibed for 24 h were incubated for 30 min at 25°C in a 50 mM sodium acetate buffer (pH 4.5) with 0.22% H2O2 and 0.05% 3-amino-9-ethylcarbazole (AEC) dissolved in N,N-dimethylformamide (Graham et al. 1965). Enzymes that were extruded during imbibition of the embryo were detected by reacting the germinator with 0.22% H2O2 and 0.05% 3-amino-9-ethylcarbazole (AEC) that was dissolved in N,N-dimethylformamide in 50 mM sodium acetate buffer (pH 4.5) for 30 min at 25°C or incubated in a solution with 0.22% of H2O2 and 10 mM of guaiacol in 50 mM phosphate buffer (pH 6.8) for 15 minutes at 25°C. Both of these reactions were carried out in the dark. For the histochemical sections, the embryos were incubated with AEC and H2O2, followed by fixation, infiltration, and cutting, and were observed with Nomarski differential interference contrast microscopy using an Axoskop Zeiss microscope. Results. There are high PODa levels at the scutellum surface that is in contact with the SE in addition to the embryonic root region (Additional file 1a). During imbibition, PODa is extruded from the embryo (Additional file 1b), confirming its activity in the apoplast via histological sectioning. A recapitulation of epidermal cell morphology after 24 hours of imbibition and papillate cell after 36 h of imbibition are shown in the Additional file 1c. Peroxidase activity at scutellar surface and extrusion to germinator. a, in situ PODa in embryo; b, PODa extruded from embryo to germinator after 24 h of imbibition; c, recapitulation of epidermal cell morphology after 24 h of imbibition and papillate cell after 36 h of imbibition. (TIFF 4 MB) [file 40064_2014_1101_MOESM1_ESM.tiff]

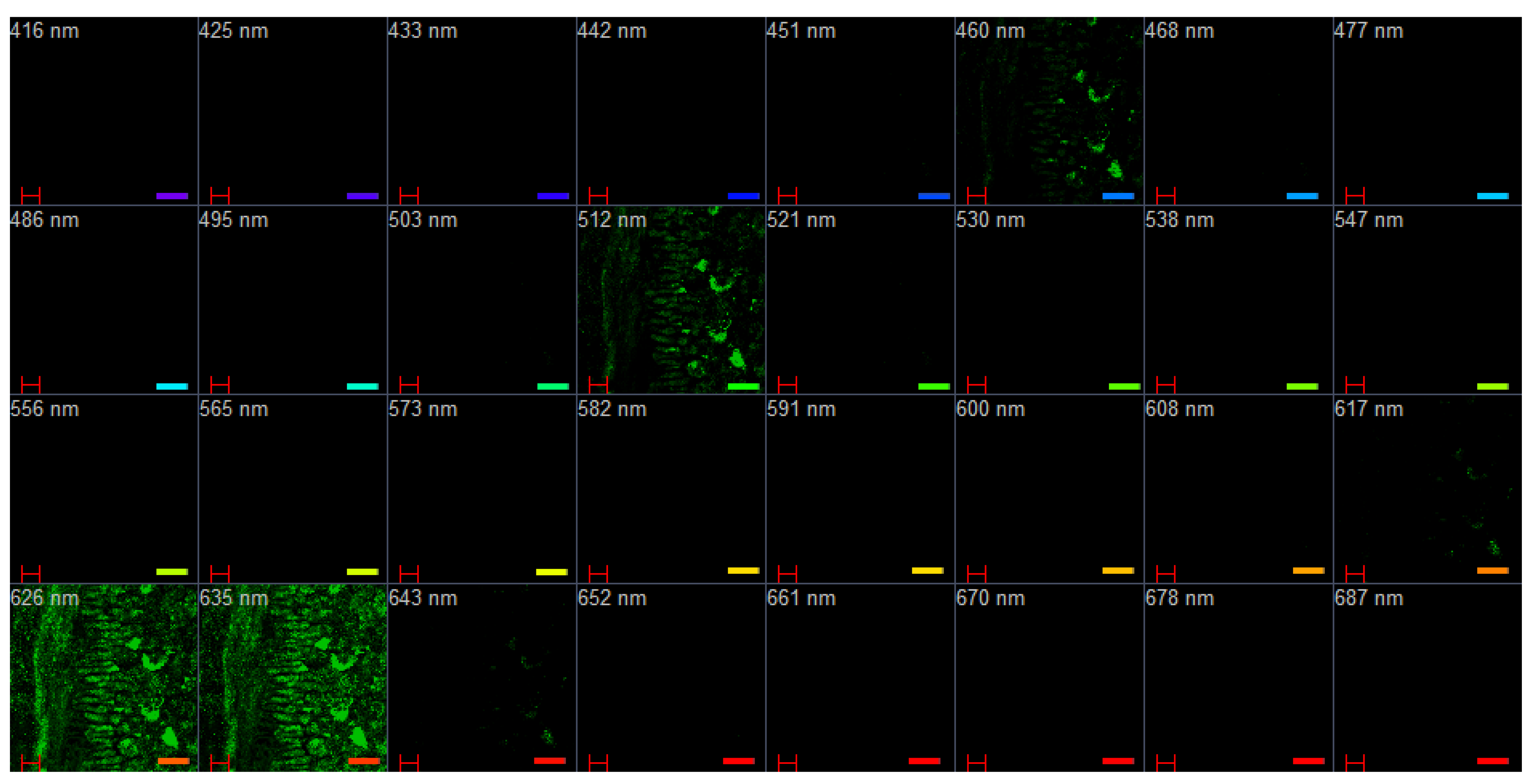

Supplement: Supplementary file 2 — Additional file 2: Emission spectrum of the scutellum at 0 h of imbibition. Embryos of dry grains were fixed in 70% ethanol at room temperature for one day, and then dehydrated, infiltrated, and embedded in paraplast. Sections of 8-μm thickness were obtained and mounted on a microscope slide cover with a gelatin film. The support media were extracted from specimens and mounted in an aqueous medium at pH 7.0, and the emissions of the visible spectrum were recorded following excitation at 405, 458, 488, 514, 561, or 633 nm with a laser from the Carl Zeiss Spectral Confocal Microscopy System Model LSM 780 NLO. Result. The emission spectrum of the scutellum at 0 h of imbibition showed 3 emission peaks: blue at 460 nm; green at 512 nm; and red between 626–635 nm. Slight emissions at 617 and 643 nm were also observed. Each box contains the emission wavelength and color corresponding to the visible spectrum. Bar: 20 μm. (TIFF 2 MB) [file 40064_2014_1101_MOESM2_ESM.tiff]
